# Screening Tool for Mental Health Problems During COVID-19 Pandemic: Psychometrics and Associations With Sex, Grieving, Contagion, and Seeking Psychological Care

**DOI:** 10.3389/fpsyg.2022.882573

**Published:** 2022-06-10

**Authors:** Silvia Morales Chainé, Rebeca Robles García, Alejandra López Montoya, Alejandro Bosch Maldonado, Ana Gisela Beristain Aguirre, Claudia Lydia Treviño Santa Cruz, Germán Palafox Palafox, Isaura Angélica Lira Chávez, Lydia Barragán Torres, María Gudelia Rangel Gómez

**Affiliations:** ^1^Facultad de Psicología, Universidad Nacional Autónoma de México, Mexico City, Mexico; ^2^Departamento de Investigaciones Sociales, Instituto Nacional de Psiquiatría “Ramón de la Fuente Muñiz”, Mexico City, Mexico; ^3^Dirección General de Atención a la Comunidad, Universidad Nacional Autónoma de México, Mexico City, Mexico; ^4^Instituto de Biotecnología, Universidad Nacional Autónoma de México, Mexico City, Mexico; ^5^Sección Mexicana de la Comisión de Salud Fronteriza México-Estados Unidos, Mexico City, Mexico

**Keywords:** stress, mental health symptoms, CFA, measurement invariance, COVID-19

## Abstract

**Background:**

The COVID-19 pandemic has created a public mental health crisis. Brief, valid electronic tools are required to evaluate mental health status, identify specific risk factors, and offer treatment when needed.

**Objective:**

To determine the construct validity, reliability, and measurement invariance of a brief screening tool for mental health symptoms by sex, loss of loved ones, personal COVID-19 status, and psychological care-seeking during the COVID-19 pandemic. Furthermore, the aim involved establishing a predictive pattern between the mental health variables.

**Method:**

A total sample of 27,320 Mexican participants, with a mean age of 32 years (*SD* = 12.24, *range* = 18–80), 67% women (*n* = 18,308), 23.10% with a loss of loved ones (*n* = 6,308), 18.3% with COVID-19 status (*n* = 5,005), and 18.40% seeking psychological care (*n* = 5,026), completed a questionnaire through a WebApp, containing socio-demographic data (sex, loss of loved ones, COVID-19 status, and psychological care-seeking) and the dimensions from the Posttraumatic Checklist, Depression-Generalized Anxiety Questionnaires, and Health Anxiety-Somatization scales. We used the confirmatory factor analysis (CFA: through maximum likelihood to continuous variable data, as an estimation method), the invariance measurement, and the structural equational modeling (SEM) to provide evidence of the construct validity of the scale and the valid path between variables. We analyzed the measurement invariance for each dimension by comparison groups to examine the extent to which the items showed comparable psychometric properties.

**Findings:**

The tool included eight dimensions: four posttraumatic stress symptoms -intrusion, avoidance, hyperactivation, and numbing, as well as depression, generalized anxiety, health anxiety, and somatization The tool’s multidimensionality, was confirmed through the CFA and SEM. The participants’ characteristics made it possible to describe the measurement invariance of scales because of the participants’ attributes. Additionally, our findings indicated that women reported high generalized anxiety, hyperactivation, and depression. Those who lost loved ones reported elevated levels of intrusion and health anxiety symptoms. Participants who reported having COVID-19 presented with high levels of generalized anxiety symptoms. Those who sought psychological care reported high levels of generalized anxiety, intrusion, hyperactivation, and health anxiety symptoms. Our findings also show that intrusion was predicted by the avoidance dimension, while health anxiety was predicted by the intrusion dimension. Generalized anxiety was predicted by the health anxiety and hyperactivation dimensions, and hyperactivation was predicted by the depression one. Depression and somatization were predicted by the health anxiety dimension. Last, numbing was predicted by the depression and avoidance dimensions.

**Discussion and Outlook:**

Our findings indicate that it was possible to validate the factor structure of posttraumatic stress symptoms and their relationship with depression, anxiety, and somatization, describing the specific bias as a function of sociodemographic COVID-19-related variables. We also describe the predictive pattern between the mental health variables. These mental health problems were identified in the community and primary health care scenarios through the CFA and the SEM, considering the PCL, depression, generalized anxiety, health anxiety, and somatization scales adapted during the COVID-19 pandemic. Therefore, future studies should describe the diagnosis of mental health disorders, assessing the cut-off points in the tool to discriminate between the presence and absence of conditions and mental health cut-off points. Community and primary care screening will lead to effective early interventions to reduce the mental health risks associated with the current pandemic.

**Limitations:**

Future studies should follow up on the results of this study and assess consistency with diagnoses of mental health disorders and evaluate the effect of remote psychological help. Moreover, in the future, researchers should monitor the process and the time that has elapsed between the occurrence of traumatic events and the development of posttraumatic stress and other mental health risks through brief electronic measurement tools such as those used in this study.

## Introduction

The risk of suffering from SARS-CoV-2 (COVID-19) began at the end of 2019 in Hubei Province, China, and spread worldwide. By 29 March 2022, over 150.4 million people had been diagnosed with COVID-19, and there had been 2.7 million deaths, with a mortality rate of 1.8%, in America alone (Panamerican Health Organization, 2022). Moreover, the risk of suffering from COVID-19 and losing loved ones to COVID-19 is associated with stress, depression, and anxiety (Rogers et al., 2020), which are not always followed by seeking psychological care.

Necho et al. (2021), summarized data on mental health symptoms from 16 studies assessing 78,225 participants. They reported 37.54% suffering from stress, 38.12% from anxiety, and 34.31% from depression and pointed out COVID-19 as a potential public mental health problem for the global community. In Mexico, Morales-Chainé et al. (2020, 2021a,2021b) reported high frequencies of stress, sadness, and anxiety symptoms according to an evaluation of 33,044 participants during the COVID-19 pandemic. These studies reported that mental health symptoms have varied due to sex, COVID-19 condition, alcohol abuse, and suffering from abuse. Measuring these mental health conditions is therefore essential.

Posttraumatic stress disorder (PTSD), which is no longer coded as an anxiety disorder in the Statistical Manual of Mental Disorders (American Psychiatric Association [APA], 2013), has been measured throughout a Checklist (PCL) developed by Weathers et al. (1994). It has been developed several tool versions -one for the military (PCL-M), one for civilians (PCL-C), and one for special populations (PCL-S; McDonald and Calhoun, 2010). Consequently, the validation of PCL-C is crucial to its remote use during the COVID-19 pandemic.

As a result of using the PCL-C, its 17-item instrument (Five-option Likert response) has been validated to assess stress in different samples, particularly the civil population (PCL-C) experiencing traumatic events. Asmundson et al. (2000), reported the PCL-C factor structure. Specifically, they reviewed 349 papers on their Confirmatory Factor Analysis (CFA) in primary care settings, using the Diagnostic and the American Psychiatric Association [APA] (2000). Researchers reported that the hierarchical model of four factors was an adequate fit model, comprising an *X*^2^(114) = 392.21, a Root Mean Square Error of Approximation *(RMSEA)* = 0.08, a Standardized Root Mean Square Residual *(SRMR)* = 0.07, a Tucker-Lewis Index *(TLI)* = 0.9, and a Comparative Fit Index (*CFI)* = 0.091. Subsequently, in McDonald and Calhoun (2010), reported the temporal stability, internal consistency, test-retest reliability, and convergent validity of the PCL-C. In Wilkins et al. (2011), after analyzing 72 papers, reported adequate fit indices indicating a satisfactory four-factor structure.

It is known that the PCL correlates moderately to firmly with other mental health symptoms such as those related to anxiety, depression, and physical functioning (McDonald and Calhoun, 2010). Elhai and Palmieri (2011) recommended analyzing both the factor structure latent variables of the PCL and the screening instruments correlating with it. These could shed light on the etiology, evolution, and treatment of PTSD and these other mental health symptoms from their early stages.

In this respect, Goldberg et al. (2017) measured depression and anxiety symptoms in 1,488 participants with a scale of 10, five-option-response items. They concluded that their tool was a valid screening instrument for depression-anxiety diagnosis in primary care settings (with 89.6% of above threshold mood or anxiety disorder diagnoses). Morales-Chainé et al. (2021a) adapted the scale with 0 to 10-option-response items. They reported that the avoidance-depression scale resulted with a Cronbach alpha of 0.73, a *X*^2^(10) = 15913.02, a *RMSEA* = 0.014, a *SRMR* = 0.005, a *TLI* = 0.999, and a *CFI* = 1. The authors also reported that the generalized anxiety scale got a Cronbach alpha of 0.93, a *X*^2^(6) = 30,032, a *RMSEA* = 0, a *SRMR* = 0, a *TLI* = 1.000, and a *CFI* = 1.

Regarding the assessment of somatization and based on a review of 31 theoretical papers, Velasco et al. (2006) have suggested that these symptoms coexisted with pathological anxiety and depression diagnoses. They have defined somatic symptoms such as those with a non-organic cause (SWOC) and signs of unjustified clinical occurrence. Velasco et al. (2006) have concluded that SWOC is associated with contextual, demographic, and individual subjectivity.

Afterward, Morales-Chainé et al. (2021b) reported a somatization scale with a Cronbach alpha of 0.96, a *X*^2^(10) = 20656.78, a *RMSEA* = 0.009, a *SRMR* = 0.002, a *TLI* = 0.999, and a *CFI* = 1. They also reported a health anxiety scale with a *X*^2^(6) = 42,994.87, a *RMSEA* = 0, a *SRMR* = 0, a *TLI* = 1, and a *CFI* = 1.

After validating the named scales, Morales-Chainé et al. (2021a) reported a predictive path between dimensions, where sadness and anxiety were associated with acute stress. Particularly, Morales-Chainé et al. (2021b), through a structured equational model (SEM) found a similar path where avoidance predicted acute stress, acute stress predicted health anxiety, health anxiety predicted generalized anxiety and somatization, and generalized anxiety/depression predicted numbing/anger.

In the context of good-fitted tools to measure mental health symptoms, Elhai and Palmieri (2011) suggested considering the moment when those instruments are administered, the sociodemographic characteristics of the population, settings, and research methods to maintain a better understanding of the symptoms and the valid factor structure of the tools. Together with the PCL-C, decisions about factor structure and the latent variables of anxiety, depression, and somatization symptoms may differ due to participants’ country, clinical setting, or demographic characteristics (Goldberg et al., 2017).

Moreover, the assessment of tools measurement invariance, suggested by McDonald and Calhoun (2010) and Elhai and Palmieri (2011), are actions that could reveal the biases between compared groups when analyzing sociodemographic variables (Millsap, 2011). Calculation of the measurement invariance could guide decision-making on risky levels of mental health that could vary because of the population’s characteristics (community vs. specialized settings), type of traumatic events, and cultural conditions (Wilkins et al., 2011).

As a tool of measurement invariance assessment (metric, strong, and strict) by comparing samples (by sex or care-seeking), the CFA generates evidence of the structural stability of the measurement. Invariance measurement is a way to establish how many of the groups-of-comparison differences and the between-symptoms predictive level result from the latent variables of interest, which could be an effect of the differences in the psychometric characteristics of the items. It is, therefore, possible to compare groups by sociodemographic or cultural factors or willingness to accept intervention (Elhai and Palmieri, 2011). The structure factor of mental health screening and its fitted model analyses are justified when researchers must work with new populations, different cultures, traumas, or novel methods, such as those used during the COVID-19 pandemic (Elhai and Palmieri, 2011). Research on the structure factor of screening tests could be linked to events during the COVID-19 pandemic, when convenience took priority over accuracy during the early stages of mental health symptoms, and when the mental-health-symptoms relationships are beneficial to understanding their progression.

Since mental health risks progress and are associated with sociodemographic conditions, it is essential to describe their relationship by analyzing measurement invariance and the scope of these comparisons with a verified structure factor test in Mexico. Accordingly, the purpose of this study was to determine the construct validity and reliability of a brief screening tool for (a) mental health symptoms; (b) comparing mental health symptoms by sex, loss of loved ones, COVID-19 status, and psychological care-seeking during the COVID-19 pandemic; through (c) examining measurement invariance of the test items between comparison groups, and (d) establishing the predictive pattern between mental health variables through the SEM.

## Method

### Design

We used a correlational study in which participants were invited to enter a programmed platform, WebApp, between 1 January and 31 August 2021. The link was available through the Mexican Health Ministry Website (announced by press conferences on the radio, television, and Internet).

Participants were asked to read the instructions. *The risk of suffering from COVID-19 is an unprecedented social condition that affects us. The current COVID-19 pandemic is a situation in which we must understand our feelings. As a result, we should see what to do about it and where to find professional help based on evidence whenever needed. We, therefore, invite you to answer the following questionnaire. You will receive feedback on your answers and counseling to cope with the emotions, thoughts, and behaviors due to the current health contingency. Your participation is voluntary, and the information you provide will be treated confidentially. Your information management will be attached to the Mexican privacy policies for personal data treatment.*

### Participants

We surveyed 27,320 persons whom participation was voluntary. Thus, sample wasn’t homogeneous. Participants were 32 years (*SD* = 12.24; range = 18–80; 10.4% of 18–19; 25.5% of 20–24; 16% of 25–29; 12.2% of 30–34; 10% of 35–39; 8% of 40–44; 6.8% of 45–49; 4.8% of 50–54; and 6.5% over 55 years), 67% were women (*n* = 18,308), 23.10% reported the loss of loved ones (*n* = 6,308), 18.30% reported COVID-19 symptoms or diagnosis (*n* = 5,005), and 18.40% were seeking remote psychological care (*n* = 5,026: see Table 1).

**TABLE 1:** Participants’ distribution by sex, loss of loved ones, COVID-19 status, and psychological care-seeking groups.

Total											
**Women**				**Men**				**Total**			
***n***		**%**		***n***		**%**		***n***		**%**	
18,308		67.00		9,012		33.00		27,320		100%	
***n***	**%**	***n***	**%**	***n***	**%**Y	***n***	**%**	***n***	**%**	***n***	**%**
					
**Non-loss of loved one**		**Loss of loved one**		**Non-loss of loved one**		**Non-loss of loved one**		**Non-loss of loved one**		**Non-loss of loved one**	
13,985	76.4	4,323	23.6	7,027	78	1,985	22	21,012	76.9	6,308	23.1

**Non-Covid-19 status**		**Covid-19 status**		**Non-Covid-19 status**		**Covid-19 status**		**Non-Covid-19 status**		**Covid-19 status**	
14,926	81.5	3,382	18.5	7,389	82	1,623	18	22,315	81.7	5,005	18.3

**Non-psychological care-seeking**		**Psychological care-seeking**		**Non-psychological care-seeking**		**Psychological care-seeking**		**Non-psychological care-seeking**		**Psychological care-seeking**	
14,685	80.2	3,623	19.8	7,609	84.4	1,403	15.6	22,294	81.6	5,026	18.4

Participants agreed to answer the survey according to the privacy policies established in the General Protection of Personal Information in Possession of Obligated Parties Act (Spanish Acronym LGPDPPSO, 2017) and the General Office of the Community Care Guidelines of the National Autonomous University of Mexico (Spanish Acronym DGACO-UNAM). Data were asymmetrically encrypted. The database was held in the official university domain, with security locks to protect the information and guarantee their management with the participants’ informed consent.

Researchers explained to participants that confidentiality would be maintained by calculating general averages in the informed consent form. Participants were told that they would be used for dissemination and epidemiological research. They had the right to decline the use of their information and withdraw from participation in the study. Incentives were not given, but immediate feedback was supplied in psychoeducational tools (infographics, videos, and Moodle ^®^ courses on COVID-19, self-care, relaxation techniques, problem-solving, and socioemotional management skills). Phone numbers were provided to obtain remote psychological care from the Health Ministry and the UNAM Services. Finally, the benefits of accessing the platforms or calling to deal with mental health conditions were described. A data section to request remote psychological care was included where participants could give their phone number or email so that they could be contacted. The protocol was approved by the Psychology College Ethics Committee on Applied Research from UNAM on 16 October 2020.

### Instruments

For this study, we used a WebApp programmed through Linux^®^, PHP^®^, HTML^®^, CSS^®^, and JavaScript^®^ software (Morales-Chainé et al., 2020, 2021a,2021b). The Cronbach alpha of the tool was 0.96. It included (1) sociodemographic and COVID-19-related variables: sex, loss of loved ones due to COVID-19, COVID-19 state (suspected or confirmed COVID-19), and remote psychological care-seeking; (2) the PCL-C test with 15 items (adapted from Weathers et al., 1994; Asmundson et al., 2000 by Morales-Chainé et al., 2020, 2021a,2021b), with 10 option responses (from zero, nothing, to 10, totally), and a four-factor structure [intrusion, with five items (e.g., *I repeatedly think or imagine I am going to get sick*), avoidance, with three items (e.g., *I try to avoid thinking, feeling, or talking about the disease*), numbing, with four items (e.g., *I have lost interest in activities I previously enjoyed*), and hyperactivation, with three items (e.g., *I find it difficult to fall or stay asleep*)]; (3) depression (Arrieta et al., 2017; Goldberg et al., 2017; Morales-Chainé et al., 2021b), consisting of 3 items with 10 response options from 0 to 10 (e.g., *I experience very little interest or pleasure in activities*); (4) Generalized Anxiety scale (Goldberg et al., 2017), comprising 5 items with 10 response options from 0 to 10 (e.g., *I have felt nervous or on edge*); (5) Health Anxiety scale (Velasco et al., 2006; Morales-Chainé et al., 2020), which has 4 items, with response options from 0 to 10 (e.g., *I feel worried about my general state of health*); and (6) Somatization scale, with four items with response options from 0 to 10 [Velasco et al., 2006; Morales-Chainé et al., 2020; e.g., *I monitor myself (self-touching, self-observation, etc.), I record what I note or feel in my body*].

### Data Analysis

We examined the multidimensionality of the scale to provide its construct-validity evidence. We run the confirmatory factor analysis (CFAs) through the maximum-likelihood to continuous-variable-data estimation method (Elhai and Palmieri, 2011).

The factors considered were an intrusion, avoidance, hyperactivation, numbing, depression, generalized anxiety, health anxiety, and somatization. The multidimensional model was adjusted, and the final items in each scale obtained standardized factor loadings above 0.4. The overall fit of the models was assessed using the chi-square goodness of fit test. Since the chi-square goodness of fit test is over-sensitive to large sample sizes, more emphasis was given to fit indices such as the CFI, TLI, RMSEA, and SRMR. Models with CFI and TLI values greater than 0.9 and RMSEA and SRMR values smaller than 0.08 and 0.06 were considered indicators of adequate data fit (Browne and Cudeck, 1993; West et al., 2012). Modification Indices (MI) were examined to determine which items needed to be correlated to get a better model adjustment.

The statistical procedure consisted of several analytical steps. Based on the sex of the participants, first, the entire sample was randomly divided into two subsamples to compare and verify the CFA results through its replication, getting the multidimensional model. Two groups resulted in 13,660 participants in sample 1 and 13,660 in sample 2 (same men-women proportion in both samples). The distribution of participants according to sex, loss of loved ones due to COVID-19, COVID-19 status, and psychological care-seeking groups for both sub-samples and the total sample is shown in Supplementary Appendix A.

The second step involved fitting the model to each of the two samples and the entire sample through the chi-square goodness of fit test, emphasizing the fit indices. The factors loading of each item and scale are shown in Supplementary Appendix B. Once we determined the final model, the third step involved calculating the reliability of the tool with their Cronbach Alpha and the correlations between scales with the Pearson analysis to identify the level of the relationship and the independence between the dimensions. Correlations are shown in Supplementary Appendix C.

The fourth step consisted of analyzing the measurement invariance for the whole sample for each dimension by comparison group (by sex, loss of loved ones, COVID-19 status, and psychological care-seeking), to examine the extent to which the items showed equivalent psychometric properties. A series of multiple-group CFA models fit the data, each with increasing equality constraints in the item parameters (Jöreskog, 1971; Sörbom, 1974; Vanderberg and Lance, 2000).

Prior, configural invariance was tested by allowing all parameters (loadings, thresholds, and unique factor variances) to be freely estimated. Next, metric invariance was assessed by constraining the item loadings to equality across comparison groups. Strong measurement invariance was tested by constraining the item thresholds to equality across comparison groups. Finally, strict measurement invariance tested equality across comparison groups in the unique factor variances. Nested models were evaluated using the chi-square test for continuous data. We also examined the CFI and TLI change from the less restricted model to the more constrained model (Δ). The more constrained model with changes in the CFI values of 0.01 or less was regarded as good (Cheug and Rensvold, 2002), and the RMSEA values of 0.015 or less were also considered acceptable. In cases where the invariance models did not fit the data, partial invariance was examined by allowing some of the item parameters to vary between groups. Modification Indices (MI) were examined to determine which item parameters needed to be freely estimated across groups. The measurement invariances were calculated for each study’s comparison group (e.g., sex). As a result of the invariance measurement, we calculated Cohen’s d, considering comparison groups’ thresholds, unique variance, and standard deviation from the fitted strict model (e.g., sex).

The fifth step examined the difference between groups with the whole sample according to the latent means of dimensions (e.g., loss of loved ones). In the final invariance model, we constrained each group’s latent variables, comparing the model’s fit with and without constraints in the means. Again, significant chi-square values, CFI values of less than 0.01, and RMSEA values differences (Δ) of less than 0.015 indicated that the constrained means model was a model with restrictions with a good fit, meaning there were no significant differences between groups.

In the sixth step, we undertook means, standard deviation, multivariate analysis, and return to Cohen’s *d* effect analysis to consider such means comparison of the dimensions with the whole sample. Finally, we integrated an overall model, including the prediction between latent variables via a chi-square test and their fit indices through structural equation modeling (SEM; Morales-Chainé et al., 2021b).

The descriptive analyses were conducted in IBM ^®^ SPSS 25 software. The confirmatory factor loading analysis, and the structural equation modeling, were conducted in RSTUDIO ^®^ 1.4.1106 through the Lavaan 0.6-9 package, ending after the necessary number of iterations to estimate the standard errors, observed information, and Hessian observed information. Specifically, through the maximum-likelihood packages, we used the Model Optimization Method, the number of free parameters, and observations to validate the models. Furthermore, we used the Model Test User Model with their test statistics, degrees of freedom, *p*-value (chi-square), and the Model Test Baseline Model packages to get the fit index.

## Finding

### Confirmatory Factorial Analyses

Results from the eight-factor model are shown in Table 2. The fits to the data in both samples were adequate, with *RMSEAs* < 0.08, *SRMRs* < 0.06, and *TLIs*, and *CFIs* > 0.9, indicating a similar factor structure between them and with the total one. Thus, a similar CFA model was obtained in the two samples and the whole one. As noted in Table 2, overall model, in sample 1, obtained a *X*^2^(406) = 20,479.87, *p* < 0.001; a *RMSEA* = 0.06; a *SRMR* = 0.049; a *CFI* = 0.928; and a *TLI* = 0.917. The overall model for sample 2 obtained a *X*^2^(406) = 23,536.61, *p* < 0.001; a *RMSEA* = 0.065; a *SRMR* = 0.042; a *CFI* = 0.936; and a *TLI* = 0.927. The overall model for the whole sample showed a *X*^2^(406) = 43,509.5, *p* < 0.001; a *RMSEA* = 0.062; a *SRMR* = 0.046; a *CFI* = 0.933; and a *TLI* = 0.923. The factor loadings from each item of the eight factors for each sample and the total one, resulting from the CFAs, are included in Supplementary Appendix B. The correlations between scales are shown in Supplementary Appendix C. In addition, Table 2 shows Cronbach’s analysis coefficients for each dimension and overall model, in both sub-samples and the total sample. Reliability values were α = 0.95 for sample 1, α = 0.97 for sample 2, and α = 0.96 for the total sample.

**TABLE 2:** Fit indices, Chi-square analysis, and Cronbach’s alpha, of the overall tool, for each sub-sample, and from the whole one.

	*X* ^2^	*df*	*p ≤*	*RMSEA*	*SRMR*	*CFI*	*TLI*	Cronbach’s alpha
**Overall CFA**								
Sample 1	20479.870	406	0.001	0.060	0.049	0.928	0.917	0.95
Sample 2	23536.610	406	0.001	0.065	0.042	0.936	0.927	0.97
Total	43509.500	406	0.001	0.062	0.046	0.933	0.923	0.96

The MI resulted in adding a correlation between the items *I repeatedly have nightmares about the disease*, and *I have unwanted physical reactions when I think about the disease (such as arrhythmia, hyperventilation, sweating)* from the intrusion dimension in the whole sample and the two subsamples. Additionally, for sample 2, specifically in the intrusion dimension, a correlation between the following items was added: *I try to avoid thinking, feeling, or talking about the disease*, and *I try to avoid looking up or referring to official information on the disease.* The MI also indicated a correlation between items *I have lost interest in activities I previously enjoyed*, and *I have felt distant from people with whom I regularly interact since the pandemic*, for the numbing dimension of sample 1 and for the total sample. Finally, the MI indicated a correlation between items *I feel worried about my general state of health*, and *I believe that I suffer from a severe physical disease (even though it hasn*’*t been confirmed)*, as well as between *I am currently worried about a certain number of physical pain spots in my body*, and *I believe I am suffering from a severe physical disease (even though it hasn*’*t been confirmed)* in the health anxiety dimension, in sample 2 and in the total sample.

### Measurement Invariance

Tables 3A–D show the results of measurement invariance models comparisons of the eight dimensions, by sex, loss-of-loved-ones, COVID-19 status, and psychological care-seeking, respectively. As expected, the difference in the chi-square test of model fit of the configural, metric, strong, and strict invariance models was significant in most comparisons due to the large sample sizes; we considered the change in CFIs and RMSEA. As in every comparison, we incorporated the correlation between the four pairs of items referred to in the CFAs section (intrusion, numbing, and health anxiety dimensions) as MI indicated. Specifically, by sex and psychological care-seeking comparison groups, correlations between the first three pairs of items were restricted to equality during the invariance measurement calculation, obtaining an adequately fitted model. For the loss of loved ones and COVID-19 status groups, we added the restricted correlations between the four pairs of items of health anxiety during the invariance measurement calculation. In Table 4, we resumed the freely estimated parameters resulting from the measurement invariance analysis. We did it to avoid overemphasizing the nuisances in the assessed groups.

**TABLE 3A:** Differences between models’ chi-squares, df, measurement invariance fit indices (configural, metric, strong, and strict), and means, by sex for all dimensions.

Models	*X* ^2^ *(df)*	*CFI*	*TLI*	*RMSEA*	*SRMR*	*ΔX^2^ (Δdf)*	*Δ X^2^*’*s p-value*	*ΔCFI*	*ΔRMSEA*	*ΔTLI*
**Intrusion**										
Configural	631.39 (8)	0.992	0.981	0.076	0.014					
Metric	655.08 (12)	0.992	0.987	0.063	0.017	23.69 (4)	0.000	0.000	−0.013	−0.006
Strong	792.55 (16)	0.990	0.988	0.060	0.020	137.47 (4)	0.000	0.002	−0.003	−0.001
Strict	1017.68 (21)	0.988	0.988	0.059	0.027	225.13 (5)	0.000	−0.002	−0.001	0.000
Strict with correlations between items	1040.49 (22)	0.987	0.989	0.058	0.027	247.94 (6)	0.000	−0.003	−0.002	−0.001

Means comparison	1416.20 (23)	0.983	0.985	0.067	0.053	375.71 (1)	0.000	0.004	0.009	0.004
**Avoidance**										
Configural										
Metric	33.44 (2)	0.998	0.995	0.034	0.014					
Strong	95.77 (4)	0.995	0.993	0.041	0.018	62.34 (2)	0.000	0.003	0.007	0.002
Strict	166.11 (7)	0.991	0.993	0.041	0.021	70.33 (3)	0.000	0.004	0	0.000
Means comparison	527.16 (8)	0.972	0.979	0.069	0.051	361.05 (1)	0.000	0.019	0.028	0.014

**Numbing**										
Configural	0.45 (2)	1.000	1.000	0.000	0.000					
Metric	21.16 (5)	1.000	0.999	0.015	0.008	20.71 (3)	0.000	0.000	0.015	0.001
Strong	157.04 (8)	0.996	0.995	0.037	0.015	135.87 (3)	0.000	0.004	0.022	0.004
Partial strong	25.01 (6)	1.000	0.999	0.015	0.008	3.85 (1)	0.050	0.000	0.000	0.000
Partial strict	95.02 (8)	0.998	0.997	0.028	0.014	70.00 (2)	0.000	−0.002	0.013	0.002
Partial strict with correlations between items	101.67 (9)	0.998	0.997	0.027	0.015	76.66 (3)	0.000	−0.002	0.012	0.002
Means comparison	214.95 (10)	0.995	0.994	0.039	0.028	113.28 (1)	0.000	0.003	0.012	0.003

**Hyperactivation**										
Configural										
Metric	7.14 (2)	1.000	1.000	0.014	0.005					
Strong	21.03 (4)	0.999	0.999	0.018	0.007	13.90 (2)	0.001	0.001	0.004	0.001
Strict	44.89 (7)	0.999	0.999	0.020	0.008	23.86 (3)	0.000	0.000	0.002	0.000
Means comparison	705.13 (8)	0.978	0.984	0.08	0.074	660.24 (1)	0.000	0.021	0.060	0.015

**Depression**										
Configural										
Metric	41.06 (2)	0.999	0.997	0.038	0.013					
Strong	88.86 (4)	0.998	0.997	0.039	0.015	47.81 (2)	0.000	0.001	0.001	0.000
Strict	274.48 (7)	0.993	0.994	0.053	0.035	185.61 (3)	0.000	−0.005	0.014	0.003
Means comparison	817.79 (8)	0.979	0.984	0.086	0.073	543.31 (1)	0.000	0.014	0.033	0.010

**Generalized anxiety**										
Configural	464.27 (10)	0.996	0.992	0.058	0.008					
Metric	486.93 (14)	0.996	0.994	0.050	0.010	22.66 (4)	0.000	0.000	−0.008	−0.002
Strong	715.39 (18)	0.994	0.993	0.053	0.015	228.46 (4)	0.000	0.002	0.003	0.001
Strict	930.92 (23)	0.992	0.993	0.054	0.017	215.53 (5)	0.000	−0.002	0.001	0.000
Means comparison	1579.62 (24)	0.987	0.989	0.069	0.068	648.70 (1)	0.000	0.005	0.015	0.004

**Health anxiety**										
Configural	173.92 (2)	0.997	0.984	0.079	0.007					
Metric	187.21 (5)	0.997	0.993	0.052	0.010	13.29 (3)	0.004	0.000	−0.027	−0.009
Partial metric	174.09 (3)	0.997	0.990	0.065	0.007	0.174 (1)	0.677	0.000	−0.014	−0.006
Partial strong	195.71 (4)	0.997	0.991	0.059	0.009	21.62 (1)	0.000	0.000	−0.006	−0.001
Partial strict	284.20 (6)	0.996	0.992	0.058	0.012	88.49 (2)	0.000	−0.001	−0.001	−0.001
Partial strict with correlations between items	288.27 (7)	0.996	0.993	0.054	0.012	92.57 (3)	0.000	−0.001	−0.005	−0.002
Means comparison	429.66 (8)	0.994	0.990	0.062	0.033	141.38 (1)	0.000	0.002	0.008	0.003

**Somatization**										
Configural	43.79 (4)	0.998	0.995	0.027	0.005					
Metric	46.38 (7)	0.998	0.997	0.020	0.007	2.59 (3)	0.460	0.000	−0.007	−0.002
Strong	174.43 (10)	0.993	0.992	0.035	0.015	128.05 (3)	0.000	0.005	0.015	0.005
Strict	202.62 (14)	0.992	0.993	0.031	0.017	28.19 (4)	0.000	−0.001	−0.004	−0.001
Means comparison	221.248 (15)	0.991	0.993	0.032	0.019	18.63 (1)	0.000	0.001	0.001	0.000

**TABLE 3B:** Differences between models’ chi-squares, df, measurement invariance fit indices (configural, metric, strong, and strict), and means, by groups with the loss of loved ones, for all dimensions.

Models	*X^2^(df)*	*CFI*	*TLI*	*RMSEA*	*SRMR*	*ΔX^2^ (Δdf)*	*Δ X^2^*’*s p-value*	*ΔCFI*	*ΔRMSEA*	*ΔTLI*
**Intrusion**										
Configural	644.58 (8)	0.992	0.980	0.076	0.015					
Metric	779.60 (12)	0.990	0.984	0.068	0.025	135.01 (4)	0.000	0.002	−0.008	−0.004
Strong	810.17 (16)	0.990	0.987	0.060	0.026	30.57 (4)	0.000	0.000	−0.008	−0.003
Strict	1304.20 (21)	0.984	0.985	0.067	0.044	494.03 (5)	2E-104	−0.006	0.007	0.002
Strict with correlation between items	1378.48 (22)	0.983	0.984	0.067	0.042	568.31 (6)	0.000	−0.007	0.007	0.003
Means comparison	2372.29 (23)	0.970	0.974	0.086	0.082	993.81 (1)	0.000	0.013	0.019	0.010

**Avoidance**										
Configural										
Metric	1.43 (2)	1.000	1.000	0.000	0.002					
Strong	54.96 (4)	0.997	0.996	0.031	0.01	53.53 (2)	0.000	0.003	0.031	0.004
Partial strong	7.41 (3)	1.000	1.000	0.010	0.005	5.98 (1)	0.014	0.000	0.010	0.000
Partial strict	251.72 (5)	0.986	0.984	0.060	0.035	244.31 (2)	0.000	0.014	0.050	0.016
Partial strict 2	11.90 (4)	1.000	0.999	0.012	0.005	4.49 (1)	0.034	0.000	0.002	0.001
Means comparison	437.12 (5)	0.976	0.972	0.080	0.046	425.22 (1)	0.000	0.024	0.068	0.027

**Numbing**										
Configural	20.90 (2)	1.000	0.997	0.026	0.003					
Metric	23.65 (5)	1.000	0.999	0.017	0.004	2.74 (3)	0.433	0.000	−0.009	−0.002
Strong	247.56 (8)	0.994	0.991	0.047	0.016	223.91 (3)	0.000	0.006	0.030	0.008
Partial strong	97.06 (7)	0.998	0.996	0.031	0.009	73.41 (2)	0.000	0.002	0.014	0.003
Partial strict	135.69 (10)	0.997	0.996	0.030	0.012	38.62 (3)	0.000	−0.001	−0.001	0.000
Partial strict with correlation between items	149.42 (11)	0.997	0.996	0.030	0.012	52.361 (4)	0.000	−0.001	−0.001	0.000
Means comparison	453.00 (12)	0.989	0.989	0.052	0.038	303.58 (1)	0.000	0.008	0.022	0.007

**Hyperactivation**										
Configural										
Metric	1.90 (2)	1.000	1.000	0.000	0.002					
Strong	59.00 (4)	0.998	0.997	0.032	0.008	57.10 (2)	0.000	0.002	0.032	0.003
Partial strong	11.98 (3)	1.000	0.999	0.015	0.004	10.08 (1)	0.002	0.000	0.015	0.001
Partial strict	50.38 (5)	0.999	0.998	0.026	0.007	38.40 (2)	0.000	−0.001	0.011	0.001
Means comparison	297.88 (6)	0.991	0.991	0.06	0.041	247.50 (1)	0.000	0.008	0.034	0.007

**Depression**										
Configural										
Metric	8.93 (2)	1.000	0.999	0.016	0.003					
Strong	43.58 (4)	0.999	0.998	0.027	0.009	34.65 (2)	0.000	0.001	0.011	0.001
Strict	76.15 (7)	0.998	0.998	0.027	0.010	32.56 (3)	0.000	−0.001	0.000	0.000
Means comparison	231.66 (8)	0.994	0.996	0.045	0.031	155.51 (1)	0.000	0.004	0.018	0.002

**Generalized anxiety**										
Configural	455.00 (10)	0.996	0.993	0.057	0.007					
Metric	471.72 (14)	0.996	0.995	0.049	0.009	16.72 (4)	0.002	0.000	−0.008	−0.002
Strong	709.86 (18)	0.994	0.994	0.053	0.015	238.14 (4)	0.000	0.002	0.004	0.001
Strict	769.33 (23)	0.994	0.995	0.049	0.016	59.47 (5)	0.000	0.000	−0.004	−0.001
Means comparison	1056.46 (24)	0.991	0.993	0.056	0.045	287.13 (1)	0.000	0.003	0.007	0.002

**Health anxiety**										
Configural	00.00 (0)	1.000	1.000	0.000	0.000					
Metric	9.16 (3)	1.000	1.000	0.012	0.004	9.16 (3)	0.027	0.000	0.012	0.000
Strong	56.68 (6)	0.999	0.998	0.025	0.007	47.53 (3)	0.000	0.001	0.013	0.002
Strict	212.64 (10)	0.997	0.996	0.039	0.015	155.95 (4)	0.000	−0.002	0.014	0.002
Strict with correlation between items	218.24 (12)	0.997	0.997	0.035	0.015	161.56 (6)	0.000	−0.002	0.010	0.001
Means comparison	679.61 (13)	0.990	0.991	0.061	0.055	461.37 (1)	0.000	0.007	0.026	0.006

**Somatization**										
Configural	35.82 (4)	0.999	0.996	0.024	0.006					
Metric	73.91 (7)	0.997	0.995	0.026	0.012	38.08 (3)	0.000	0.002	0.002	0.001
Strong	180.10 (10)	0.992	0.991	0.035	0.017	106.19 (3)	0.000	0.005	0.009	0.004
Strict	456.88 (14)	0.980	0.983	0.048	0.031	276.78 (4)	0.000	−0.012	0.013	0.008
Partial strict	267.28 (13)	0.989	0.990	0.038	0.021	87.18 (3)	0.000	−0.003	0.003	0.001
Means comparison	645.53 (14)	0.972	0.976	0.057	0.045	378.25 (1)	0.000	0.017	0.019	0.014

**TABLE 3C:** Differences between models’ chi-squares, df, measurement invariance fit indices (configural, metric, strong, and strict), and means, per COVID-19 condition, for all dimensions.

Models	*X^2^(df)*	*CFI*	*TLI*	*RMSEA*	*SRMR*	*ΔX^2^ (Δdf)*	*Δ X^2^*’*s p-value*	*ΔCFI*	*ΔRMSEA*	*ΔTLI*
**Intrusion**										
Configural	689.71 (8)	0.991	0.978	0.079	0.015					
Metric	865.09 (12)	0.989	0.982	0.072	0.026	175.38 (4)	0.000	0.002	−0.007	−0.004
Strong	995.33 (16)	0.987	0.984	0.067	0.028	130.24 (4)	0.000	0.002	−0.005	−0.002
Strict	1942.93 (21)	0.975	0.977	0.082	0.053	947.60 (5)	1E-202	−0.012	0.015	0.007
Partial strict	1320.00 (20)	0.983	0.983	0.069	0.038	324.68 (4)	5E-69	−0.004	0.002	0.001
Partial strict with correlation between items	1320.38 (21)	0.983	0.984	0.067	0.039	325.05 (5)	0.000	−0.004	0.000	0.000
Means comparison	2726.06 (22)	0.965	0.968	0.095	0.091	1405.68 (1)	0.000	0.018	0.028	0.016

**Avoidance**										
Configural										
Metric	22.83 (2)	0.999	0.997	0.028	0.009					
Strong	99.31 (4)	0.995	0.992	0.042	0.016	76.47 (2)	0.000	0.004	0.014	0.005
Strict	593.92 (7)	0.967	0.972	0.078	0.050	494.61 (3)	0.000	0.028	0.036	0.020
Partial strict	135.99 (6)	0.993	0.993	0.040	0.016	36.68 (2)	0.000	−0.002	−0.002	−0.001
Means comparison	609.39 (7)	0.967	0.971	0.079	0.050	473.40 (1)	0.000	0.026	0.039	0.022

**Numbing**										
Configural	15.03 (2)	1.000	0.998	0.022	0.002					
Metric	22.22 (5)	1.000	0.999	0.016	0.004	7.19 (3)	0.066	0.000	−0.006	−0.001
Strong	279.42 (8)	0.994	0.990	0.050	0.016	257.20 (3)	0.000	0.006	0.034	0.009
Partial strong	98.07 (7)	0.998	0.996	0.031	0.009	75.85 (2)	0.000	0.002	0.015	0.003
Partial strict	133.28 (10)	0.997	0.996	0.030	0.012	35.21 (3)	0.000	−0.001	−0.001	0.000
Partial strict with correlation between items	142.02 (11)	0.997	0.997	0.030	0.012	43.95 (4)	0.000	−0.001	−0.001	−0.001
Means comparison	423.27 (12)	0.990	0.990	0.050	0.034	281.24 (1)	0.000	0.007	0.020	0.007

**Hyperactivation**										
Configural										
Metric	2.99 (2)	1.000	1.000	0.006	0.003					
Strong	90.09 (4)	0.997	0.996	0.040	0.010	87.10 (2)	0.000	0.003	0.034	0.004
Partial strong	3.00 (3)	1.000	1.000	0.000	0.003	0.00 (1)	0.944	0.000	−0.006	0.000
Partial strict	4.85 (4)	1.000	1.000	0.004	0.003	1.86 (1)	0.173	0.000	0.004	0.000
Means comparison	234.81 (5)	0.993	0.992	0.058	0.035	229.96 (1)	0.000	0.007	0.054	0.008

**Depression**										
Configural										
Metric	10.03 (2)	1.000	0.999	0.017	0.004					
Strong	69.92 (4)	0.998	0.997	0.035	0.011	59.89 (2)	0.000	0.002	0.018	0.002
Partial strong	13.61 (3)	1.000	0.999	0.016	0.004	3.58 (1)	0.058	0.000	−0.001	0.000
Partial strict	24.80 (5)	0.999	0.999	0.017	0.004	11.19 (2)	0.004	−0.001	0.001	0.000
Means comparison	249.19 (6)	0.994	0.994	0.054	0.035	224.40 (1)	0.000	0.005	0.037	0.005

**Generalized anxiety**										
Configural	470.56 (10)	0.996	0.992	0.058	0.007					
Metric	513.92 (14)	0.996	0.994	0.051	0.011	43.36 (4)	0.000	0.000	−0.007	−0.002
Strong	728.15 (18)	0.994	0.993	0.054	0.015	214.23 (4)	0.000	0.002	0.003	0.001
Strict	767.63 (23)	0.994	0.995	0.049	0.016	39.48 (5)	0.000	0.000	−0.005	−0.002
Means comparison	1349.98 (24)	0.989	0.991	0.064	0.058	582.35 (1)	0.000	0.005	0.015	0.004

**Health anxiety**										
Configural	0.00 (0)	1.000	1.000	0.000	0.000					
Metric	57.00 (3)	0.999	0.997	0.036	0.012	57.00 (3)	0.000	0.001	0.036	0.003
Partial metric	0.723 (1)	1.000	1.000	0.000	0.001	0.723 (1)	0.395	0.000	0.000	0.000
Partial strong	0.723 (1)	1.000	1.000	0.000	0.001	000.00 (0)	0.000	0.000	0.000	0.000
Partial strict	1.62 (2)	1.000	1.000	0.000	0.001	0.894 (1)	0.344	0.000	0.000	0.000
Partial strict with correlation between items	15.49 (4)	1.000	0.999	0.015	0.002	14.77 (3)	0.002	0.000	0.015	0.001
Means comparison	931.94 (5)	0.986	0.865	0.116	0.063	916.44 (1)	0.000	0.014	0.101	0.134

**Somatization**										
Configural	35.81 (4)	0.999	0.996	0.024	0.006					
Metric	131.52 (7)	0.994	0.990	0.036	0.016	95.72 (3)	0.000	0.005	0.012	0.006
Strong	322.88 (10)	0.986	0.983	0.048	0.023	191.35 (3)	0.000	0.008	0.012	0.007
Strict	789.25 (14)	0.965	0.970	0.064	0.040	466.37 (4)	0.000	−0.021	0.016	0.013
Partial strict	508.21 (13)	0.978	0.979	0.053	0.026	185.34 (3)	0.000	−0.008	0.005	0.004
Means comparison	1222.10 (14)	0.945	0.953	0.079	0.057	713.89 (1)	0.000	0.033	0.026	0.026

**TABLE 3D:** Differences between models’ chi-squares, df, measurement invariance fit indices (configural, metric, strong, and strict), and means per psychological care-seeking condition, for all dimensions.

Models	*X^2^(df)*	*CFI*	*TLI*	*RMSEA*	*SRMR*	*ΔX^2^ (Δdf)*	*Δ X^2^*’*s p-value*	*ΔCFI*	*ΔRMSEA*	*ΔTLI*
**Intrusion**										
Configural	638.60 (8)	0.992	0.980	0.076	0.015					
Metric	768.77 (12)	0.991	0.984	0.068	0.024	130.16 (4)	0.000	0.001	−0.008	−0.004
Strong	868.88 (16)	0.989	0.987	0.062	0.025	100.11 (4)	0.000	0.002	−0.006	−0.003
Strict	1099.79 (21)	0.987	0.987	0.061	0.034	230.91 (5)	7E-48	−0.002	−0.001	0.000
Strict with correlations between items	1105.41 (22)	0.987	0.988	0.060	0.034	236.52 (6)	0.000	−0.002	−0.002	−0.001
Means comparison	1445.23 (23)	0.982	0.985	0.067	0.051	339.82 (1)	0.000	0.005	0.007	0.003

**Avoidance**										
Configural										
Metric	2.27 (2)	1.000	1.000	0.003	0.003					
Strong	31.47 (4)	0.998	0.998	0.022	0.006	29.20 (2)	0.000	0.002	0.019	0.002
Partial strong	2.33 (3)	1.000	1.000	0.000	0.003	0.06 (1)	0.810	0.000	−0.003	0.000
Partial strict	2.65 (4)	1.000	1.000	0.000	0.003	0.33 (1)	0.567	0.000	0.000	0.000
Means comparison	228.28 (5)	0.988	0.985	0.057	0.030	225.62 (1)	0.000	0.012	0.057	0.015

**Numbing**										
Configural	3.27 (2)	1.000	1.000	0.007	0.001					
Metric	124.42 (5)	0.997	0.993	0.042	0.015	121.15 (3)	0.000	0.003	0.035	0.007
Partial metric	29.29 (4)	0.999	0.998	0.022	0.008	26.02 (2)	0.000	0.001	0.015	0.002
Partial strong	73.02 (6)	0.998	0.997	0.029	0.012	43.73 (2)	0.000	0.001	0.007	0.001
Partial strict	100.35 (8)	0.998	0.997	0.029	0.012	27.32 (2)	0.000	0.000	0.000	0.000
Partial strict with correlations between items	144.83 (9)	0.997	0.996	0.033	0.013	71.81 (3)	0.000	−0.001	0.004	0.001
Means comparison	1766.95 (10)	0.957	0.948	0.113	0.086	1622.12 (1)	0.000	0.040	0.080	0.048

**Hyperactivation**										
Configural										
Metric	1.435 (2)	1.000	1.000	0.000	0.002					
Strong	15.22 (4)	1.000	0.999	0.014	0.005	13.78 (2)	0.001	0.000	0.014	0.001
Strict	41.81 (7)	0.999	0.999	0.019	0.009	26.59 (3)	0.000	−0.001	0.005	0.000
Means comparison	2198.75 (8)	0.928	0.946	0.142	0.125	2156.94 (1)	0.000	0.071	0.123	0.053

**Depression**										
Configural										
Metric	188.94 (2)	0.995	0.985	0.083	0.022					
Partial metric	14.02 (1)	1.000	0.998	0.031	0.004					
Partial strong	27.16 (2)	0.999	0.998	0.030	0.004	13.14 (1)	0.000	0.001	−0.001	0.000
Partial strict	67.27 (4)	0.998	0.997	0.034	0.009	40.11 (2)	0.000	−0.001	0.004	0.001
Means comparison	2684.14 (5)	0.927	0.912	0.198	0.137	2616.86 (1)	0.000	0.071	0.164	0.085

**Generalized anxiety**										
Configural	465.67 (10)	0.996	0.992	0.058	0.008					
Metric	638.19 (14)	0.994	0.992	0.057	0.018	172.51 (4)	0.000	0.002	−0.001	0.000
Strong	737.39 (18)	0.994	0.993	0.054	0.020	99.21 (4)	0.000	0.000	−0.003	−0.001
Strict	937.57 (23)	0.992	0.993	0.054	0.024	200.17 (5)	0.000	−0.002	0.000	0.000
Means comparison	3433.32 (24)	0.970	0.975	0.102	0.133	2495.75 (1)	0.000	0.022	0.048	0.018

**Health anxiety**										
Configural	171.53 (2)	0.997	0.984	0.079	0.007					
Metric	378.19 (5)	0.994	0.986	0.074	0.024	206.67 (3)	0.000	0.003	−0.005	−0.002
Strong	624.85 (8)	0.991	0.986	0.075	0.025	246.66 (3)	0.000	0.003	0.001	0.000
Strict	891.71 (12)	0.987	0.987	0.073	0.029	266.86 (4)	0.000	−0.004	−0.002	−0.001
Strict with correlations between items	898.73 (13)	0.986	0.988	0.071	0.030	273.88 (5)	0.000	−0.005	−0.004	−0.002
Means comparison	1619.58 (14)	0.976	0.979	0.092	0.068	720.85 (1)	0.000	0.010	0.021	0.009

**Somatization**										
Configural	34.97 (4)	0.999	0.996	0.024	0.005					
Metric	58.78 (7)	0.998	0.996	0.023	0.009	23.81 (3)	0.000	0.001	−0.001	0.000
Strong	155.55 (10)	0.994	0.992	0.033	0.012	96.77 (3)	0.000	0.004	0.010	0.004
Strict	313.57 (14)	0.987	0.989	0.040	0.018	158.03 (4)	0.000	−0.007	0.007	0.003
Means comparison	535.45 (15)	0.977	0.982	0.05	0.033	221.88 (1)	0.000	0.010	0.010	0.007

**TABLE 4:** Resulting freely estimated loadings, thresholds, and unique factor variances from partial measurement invariances, for all items in each dimension by comparison group.

Items	Comparison groups			
	Sex	Loss of loved one	COVID-19 status	Psychological care-seeking
**Intrusion**				
B1. I repeatedly think or imagine I am going to get sick.				
B2. I repeatedly have nightmares about the disease.			Unique factor variances	
B4. I feel uneasy when people talk about the disease.				
B5. I have unwanted physical reactions when I think about the disease (e.g., arrhythmia, hyperventilation, sweating).				
D5. I feel scared of the risk of getting infected.				
**Avoiding**				
C1. I try to avoid thinking, feeling, or talking about the disease.				
C2. I try to avoid looking up or referring to official information on the disease.		Thresholds and unique factor variances		Thresholds and unique factor variances
C3. I have trouble remembering the recommendations issued by the authorities regarding the pandemic.		Unique factor variances		
**Numbing**				
C4. I have lost interest in activities that I previously enjoyed.	Thresholds and unique factor variances			
C5. I have felt distant from people with whom I regularly interact since the beginning of the pandemic.				Loadings, thresholds, and unique factor variances
C6. I struggle to care about my loved ones.				Unique factor variances
C7. I feel that my future is uncertain due to the disease.	Thresholds and unique factor variances			
**Hyperactivation**				
D1. I find it difficult to fall or stay asleep.			Thresholds and unique factor variances	
D2. I feel angry.			Unique factor variances	
D3. I find it difficult to pay attention.		Thresholds and unique factor variances		
**Depression**				
I want to hurt myself.			Thresholds and unique factor variances	Loadings, thresholds, and unique factor variances
Dep2-Goldberg. R31. I feel little interest or pleasure in activities.				
Dep1-Goldberg. R32. I have felt down, depressed, or hopeless.				
**Generalized anxiety**				
AnsG1. I have felt nervous or on edge.				
AnsG2. I have felt unable to control my worrying.				
AnsG3. I have felt so restless it was hard to keep still.				
AnsG4. I have had trouble relaxing.				
AnsG5. I have felt afraid something awful could happen.				
**Health anxiety**				
I feel worried about my general state of health.	Loadings, thresholds, and unique factor variances		Loadings, thresholds, and unique factor variances	
I am currently worried about a certain number of physical pain spots in my body.			Thresholds and unique factor variances	
It scare me that I may have any severe physical disease.				
I believe I am suffering from a severe physical disease (even though it has not been confirmed).			Loadings, thresholds, and unique factor variances	
**Somatization**				
I monitor myself (self-touching, self-observing, etc.), recording what I note or feel in my body.				
I read (or I am interested in TV or radio shows) about severe physical disease.				
I talk to my family and friends about my physical pain spots.				
I feel like staying in bed, take my temperature, take my pulse, change my diet and take my meds; even though, they have not been prescribed by a doctor).		Unique factor variances		

Given that the change differences (Δ) between the measurement invariance models are smaller than 0.01 for the *CFIs* and smaller than 0.015 for the *RMSEAs*, Table 3A shows that the intrusion, avoidance, hyperactivation, depression, generalized anxiety, and somatization dimensions obtained a measurement invariance between sex groups. The numbing and health anxiety dimensions obtained a partial measurement invariance between them. Additionally, Table 3A shows that restricted means models of intrusion, numbing, generalized anxiety, health anxiety, and somatization, compared to those when means were freely estimated by sex, resulted in changes that were smaller than 0.01 for the *CFIs* and smaller than 0.015 for the *RMSEAs.*

Table 3B shows that intrusion, depression, generalized, and health anxiety dimensions obtained a measurement invariance by reporting the loss of a loved one condition. The avoidance, numbing, hyperactivation, and somatization dimensions obtained a partial measurement invariance. Moreover, Table 3B shows that restricted means models of generalized anxiety, compared to those when means were freely estimated by the loss of a loved one, resulted in changes smaller than 0.01 for the *CFIs* and smaller than 0.015 for the *RMSEAs*.

Table 3C shows that the generalized anxiety dimension obtained a measurement invariance by the COVID-19-condition. The remaining dimensions obtained a partial measurement invariance. Table 3C also shows that the restricted means model of generalized anxiety dimension, compared to the freely estimated means one, by the COVID-19-condition, resulted in changes smaller than 0.01 for the *CFIs* and smaller than 0.015 for the *RMSEAs*.

Table 3D shows that the intrusion, hyperactivation, generalized, health anxiety, and somatization dimensions obtained a measurement invariance by psychological care-seeking condition. The avoidance, numbing, and depression dimensions obtained a partial measurement invariance. Table 3D also shows that the restricted means models of the intrusion, and somatization dimensions, compared to those where the psychological care-seeking condition freely estimated means, resulted in changes smaller than 0.01 for the *CFIs* and smaller than 0.015 for the *RMSEAs*.

Table 4 resumed the freely estimated parameters resulting from partial measurement invariances, for all items, by dimension and group. Regarding the intrusion dimension and to prevent nuisances from being overemphasized in the fit models, we freely estimated the unique factor variances of the item *I repeatedly have nightmares about the disease* by COVID-19 status.

For the avoidance dimension, we freely estimated the thresholds and unique factor variances of the item *I try to avoid looking up or referring to official information on the disease by* loss of loved ones and psychological care-seeking groups. Also, we freely estimated the unique factor variances of the item *I have trouble remembering the recommendations issued by the authorities regarding the pandemic* by loss of loved ones, COVID-19 status, and psychological care-seeking groups.

As for the numbing dimension, we freely estimated thresholds and unique factor variances of the item *I have lost interest in activities I previously enjoyed* by sex group. We also freely estimated the loadings, thresholds, and unique factor variances of the item *I have felt distant from people with whom I regularly interact since the beginning of the pandemic* by the psychological care-seeking group. Additionally, we freely estimated the unique factor variances of the item *I struggle to care about my loved ones* by psychological care-seeking groups. Lastly, we freely estimated thresholds and unique factor variances of the item *I feel my future is uncertain because of the disease* by sex, loss of loved ones, and COVID-19 status groups.

Regarding the hyperactivation dimension, we freely estimated the thresholds, and unique factor variances of the item *I find difficult to fall or stay asleep*, and the unique factor variances of the item *I feel angry*, both for COVID-19-status groups. We also freely estimated thresholds and unique factor variances of the item *I find it difficult to pay attention* to the loss of a loved one group.

For the depression dimension, we freely estimated thresholds and unique factor variances of the item *I feel like doing things to hurt myself* by COVID-19-status, loadings, thresholds, and unique factor variances of the same item for psychological care-seeking groups.

Regarding the health anxiety dimension, we freely estimated the loadings, thresholds, and unique factor variances of the item *I feel worried about my general state of health* per sex and COVID-19 status groups. We freely estimated the loadings, thresholds, and unique factor variances of the item *I am currently worried about a certain number of physical pain spots in my body* by sex and the thresholds and unique factor variances of the same item by per COVID-19 status groups. Lastly, we freely estimated the loadings, thresholds, and unique factor variances of the item *I believe I am suffering from a severe physical disease (even though it hasn*’*t been confirmed)* by COVID-19 status groups.

For the somatization dimension, we freely estimated the unique factor variances of the item *I choose to stay in bed, take my temperature, take my pulse, change my diet and take meds, etc. (even though they had not been prescribed by a physician)* by loss of loved ones, and COVID-19 status groups.

Our findings on the fit models suggest that all dimensions can be used to compare means between comparison groups, considering the specifically structured bias of the items.

### Comparison Groups Means

Table 5 shows the mean (*M)* for all dimensions by sex, loss of loved ones, COVID-19 status, psychological care-seeking, *F* values, degrees of freedom, *p*-values from the multivariate analyses, and the Cohen *d* effect size from invariance measurement. Despite the high mean generalized anxiety for women, restricted means models and Cohen-d analysis suggested a moderate difference for men (*d* = −0.341). Participants’ sex had a low effect on intrusion, avoidance, numbing, health anxiety, and somatization means (*d* = −0.263, *d* = −0.27, *d* = −0.282, *d* = −0.169, and *d* = −0.064, respectively). For the remaining dimensions, based on the freely estimated means models, the Cohen’s *d* size effect analyses indicated moderate differences between hyperactivation means (*d* = −0.364) and depression (*d* = −0.318) between these groups.

**TABLE 5:** Dimensions means by sex, loss of loved ones, COVID-19 status, psychological care-seeking groups, F, df, *p*-values, from the multi-variate analyses, and Cohen’s d effect size from invariance measurement.

Dimension	Men		Women		ANOVA		*Cohen’s d*
	*M*	*SD*	*M*	*SD*	*F* (1, 27,318)	*p<*	
Intrusion	31.56	27.41	38.09	27.95	333.52	0.001	−0.263
Avoidance	24.05	23.77	29.22	24.76	270.01	0.001	−0.270
Numbing	42.66	31.35	48.84	30.38	244.56	0.001	−0.282
Hyperactivation	43.93	32.82	54.52	31.99	650.52	0.001	−0.364
Depression	35.84	31.13	44.70	31.53	481.74	0.001	−0.318
Gen. anxiety	42.34	34.44	53.42	33.57	646.69	0.001	−0.341
Health anxiety	36.61	31.14	42.78	31.60	232.27	0.001	−0.169
Somatization	28.02	24.35	29.50	24.40	22.30	0.001	−0.064

**Dimension**	**Non-loss of loved one**		**Loss of loved one**		**ANOVA**		***Cohen***’***s d***
	***M***	***SD***	***M***	***SD***	***F* (1, 36,809)**	***p*-value**	

Intrusion	32.96	27.07	45.84	28.53	1070.08	0.001	−0.492
Avoidance	25.81	24.11	33.19	25.18	445.61	0.001	−0.321
Numbing	44.93	30.96	53.02	29.61	338.04	0.001	−0.261
Hyperactivation	49.46	32.95	56.24	31.06	211.13	0.001	−0.219
Depression	40.67	31.98	45.46	30.35	111.20	0.001	−0.186
Gen. anxiety	47.76	34.41	56.41	32.87	312.4	0.001	−0.249
Health anxiety	38.46	31.21	48.36	31.61	485.68	0.001	−0.324
Somatization	27.31	23.57	34.68	26.17	449.82	0.001	−0.333

**Dimension**	**Non-COVID-19 status**		**COVID-19 status**		**ANOVA**		***Cohen***’***s d***
	***M***	***SD***	***M***	***SD***	***F* (1, 36,809)**	***p*-value**	

Intrusion	32.73	26.71	50.24	28.82	1704.44	0.001	−0.658
Avoidance	25.79	23.91	35.19	25.92	612.18	0.001	−0.397
Numbing	45.24	30.94	53.76	29.43	315.51	0.001	−0.274
Hyperactivation	49.38	32.91	58.35	30.37	312.29	0.001	−0.260
Depression	40.71	31.95	46.54	29.97	139.27	0.001	−0.234
Gen. anxiety	47.38	34.25	60.38	32.22	601.81	0.001	−0.388
Health anxiety	37.50	30.70	55.22	31.38	1351.36	0.001	−0.576
Somatization	26.87	23.28	38.55	26.81	970.11	0.001	−0.514

**Dimension**	**Non-psychological care-seeking**		**Psychological care-seeking**		**ANOVA**		***Cohen***’***s d***
	***M***	***SD***	***M***	***SD***	***F* (1, 36,809)**	***p*-value**	

Intrusion	34.35	27.54	42.99	28.61	398.23	0.001	−0.307
Avoidance	26.43	24.11	32.29	25.93	235.18	0.001	−0.253
Numbing	43.63	30.62	60.87	27.74	1344.86	0.001	−0.613
Hyperactivation	47.01	32.46	68.84	27.05	1965.333	0.001	−0.807
Depression	37.28	30.68	61.71	28.11	2678.83	0.001	−0.852
Gen. anxiety	45.28	33.88	69.64	28.34	2243.46	0.001	−0.823
Health anxiety	38.30	31.18	51.58	31.06	744.32	0.001	−0.449
Somatization	27.96	24.16	33.69	24.85	227.87	0.001	−0.272

**Dimension**	**Total**						
	***M***				***SD***		

Intrusion	35.94				27.94		
Avoidance	27.51				24.56		
Numbing	46.80				30.84		
Hyperactivation	51.03				32.65		
Depression	41.78				31.67		
Gen. anxiety	49.76				34.26		
Health anxiety	40.74				31.58		
Somatization	29.01				24.39		

Once again, although participants who reported losing loved ones obtained high means for numbing, hyperactivation, and generalized anxiety (*M* > 0.05), it is essential to consider the restricted means models fit and the Cohen-d analysis results. Our findings suggest that the difference in generalized anxiety means between those who reported the loss of loved ones and those who did not was low (*d* = −0.249). This comparison can therefore be made because of a good dimension factor structure. For the remaining dimensions, based on the freely estimated means models, the Cohen’s *d* size effect analyses indicated minimal differences for the numbing (*d* = −0.261), hyperactivation (*d* = −0.219), and depression (*d* = −0.186) dimensions means between comparison groups. Furthermore, from freely estimating parameters, our results showed moderate differences between groups for the intrusion (*d* = −0.492), avoidance (*d* = −0.321), health anxiety (*d* = −0.324), and somatization (*d* = −0.333) dimension means.

Based on the restricted mean models, our results suggest that the generalized anxiety dimension was moderately different between those who reported COVID-19 status and those who did not (*d* = −0.388). Regarding the freely estimated means models, we also found low effects for the numbing (*d* = −0.274), hyperactivation (*d* = −0.26), and depression (*d* = −0.243) dimension means between these groups. Our results also showed moderate differences between the intrusion (*d* = −0.658), avoidance (*d* = −0.393), health anxiety (*d* = −0.576), and somatization (*d* = −0.514) dimension means between groups.

For the restricted means models, our findings suggest that somatization had mildly significant effects (*d* = −0.272), and that intrusion had moderately significant effects (*d* = −0.307), due to the psychological care-seeking condition. For the free means fit models, the Cohen’s *d* size effect analyses indicated small differences for the avoidance (*d* = −0.253) dimension means between those who sought psychological care and those who did not. Our results also showed moderate differences between groups for the numbing (*d* = −0.613), and health anxiety (*d* = −0.449) dimensions means. Moreover, results showed large differences between group means for the hyperactivation (*d* = −0.807), depression (*d* = −0.852), and generalized anxiety (*d* = −0.823) dimensions.

### Structural Equation Modeling

**FIGURE 1:**
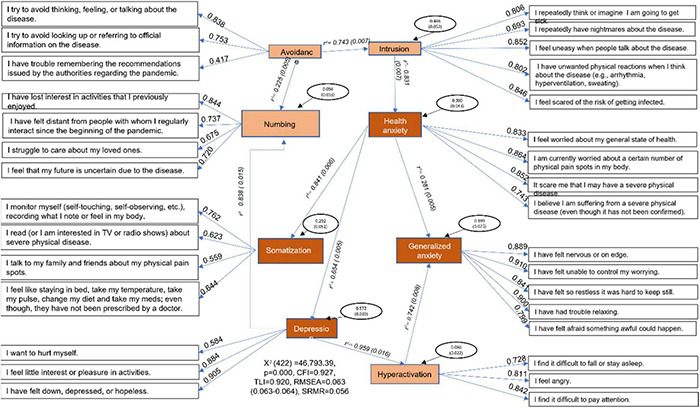
Latent variables from the structured equation model, their factor loadings, regression coefficients, residual variances, Chi-square, and fit indices.

Figure 1 shows the resulting structural equation modeling (SEM). As latent variables, the model included intrusion, avoidance, numbing, hyperactivation, depression, generalized anxiety, health anxiety, and somatization. Figure 1 shows the group of items for each latent variable, their factorial loads, the regression coefficients, and their residuals. The fit model resulted from 95 iterations with 74 parameters [*X*^2^(422) = 46,793.39, *p* < 0.001], with a *CFI* = 0.927, a *TLC* = 0.92, an *RMSEA* = 0.063 (0.063–0.064), and an *SRMR* = 0.056. Note that the difference in the free-degrees between CFA and SEM resulted from the added parameters of the SEM’s model—latent variables relationships. Consequently, our results indicated that the intrusion latent variable was predicted by the avoidance one (*R*^2^ = 0.743). Health anxiety was predicted by the intrusion latent variable (*R*^2^ = 0.831). Generalized anxiety was predicted by health anxiety (*R*^2^ = 0.281) and hyperactivation (*R*^2^ = 0.742). The hyperactivation dimension was predicted by the depression latent variable (*R*^2^ = 0.959). Depression and somatization were predicted by health anxiety (*R*^2^ = 0.654 and *R*^2^ = 0.841, respectively). Finally, in the SEM, the numbing latent variable was predicted by depression and avoidance (*R*^2^ = 0.838, and *R*^2^ = 0.225, respectively).

## Discussion

This study aimed to determine the construct validity, reliability, and measurement invariance of a brief screening tool for mental health symptoms by sex, loss of loved ones, personal COVID-19-status, and psychological care-seeking during the COVID-19 pandemic. Furthermore, the aim involved establishing a predictive pattern between the mental health variables. Our findings suggest that it was possible to validate the mental health dimensions assessed throughout a WebApp, with a fit model replication by the CFA with two samples of Mexican participants. Dimensions, in general, were constituted by factorial loadings over 0.4. Our findings indicated that we obtained a multidimensional, eight-scale instrument for the evaluation of stress (PCL-C), depression, generalized anxiety, health anxiety, and somatization based on the CFA procedures.

Four latent variables were included in the PCL-C adapted to the COVID-19 pandemic—intrusion, avoidance, hyperactivation, and numbing dimensions (Weathers et al., 1994; Morales-Chainé et al., 2020, 2021a,2021b). Findings also suggested latent variables of depression, generalized anxiety (Goldberg et al., 2017), health anxiety, and somatization (Velasco et al., 2006; Morales-Chainé et al., 2020, 2021a,2021b). Although all the dimensions were related, they were regarded as independent.

Our findings suggested that PCL-C (adapted from Weathers et al., 1994), depression, generalized anxiety (adapted from Goldberg et al., 2017), health anxiety, and somatization scales (from Velasco et al., 2006) were validated by their scheduled remote application during the COVID-19 pandemic for Mexicans. Specifically, CFA yielded a model with the goodness of fit in eight dimensions, replicated with two samples, using the Chi-square, *CFI*, *TLI*, *RMSEA*, and *SRMR* good indexes procedure (Browne and Cudeck, 1993; West et al., 2012). Thus, dimensions resulting from structural factorial analysis -intrusion, avoidance, hyperactivation, numbing (from PCL-C), depression, generalized anxiety, health anxiety, and somatization, could screen for mental health risks in the civil population experiencing events related to the COVID-19 pandemic.

Moreover, findings of invariance measurement (Millsap, 2011) enabled us to analyze and define a comparison procedure to screen for mental health symptoms, using the instrument’s factor structure. Importantly our findings suggest that is possible to compare generalized anxiety between all the groups in the study. The intrusion means were compared between the sex, loss of loved ones, and psychological care-seeking variables. Avoidance means were comparable between sex groups. Numbing means were comparable between those reporting loss of loved ones or COVID-19-status groups. Hyperactivation means were comparable between sex and those seeking remote psychological care groups. Depression means were comparable to the group of sex and loss of loved ones. Health anxiety means were comparable between loss of loved ones and psychological care-seeking groups. And somatization means were comparable between sex and psychological care-seeking groups.

Consequently, the results of the measurement invariance showed that women reported high levels of generalized anxiety, hyperactivation, and depression. Those who had lost a loved one reported a high level of intrusion and health anxiety symptoms. Participants who reported COVID-19 status also reported high levels of generalized anxiety symptoms. These findings are very similar to those reported by Morales-Chainé et al. (2021b). Moreover, in the present study, we also found that those who seek psychological care reported high levels of generalized anxiety, intrusion, hyperactivation, and health anxiety symptoms.

Given the freely estimated loadings, thresholds, and unique factor variances, it was possible to match the remaining dimensions and comparison groups considering the bias in the reviewed items. As Morales-Chainé et al. (2021b) noted, our findings suggest that those losing loved ones reported high levels of avoidance and somatization symptoms. However, the current study suggests considering the bias created by *looking up, referring to official information on the disease, not remembering the recommendations issued by the authorities regarding the pandemic*, and *feeling like staying in bed, taking their temperature, taking their pulse, changing their diet, taking meds, etc. (even though they had not been prescribed by a physician).* Despite high avoidance and somatization of losing someone, further research should analyze the variables originating from such psychometric bias.

Moreover, our findings indicate that those suspected of being infected by COVID-19 reported high levels of intrusion, avoidance, health anxiety, and somatization, including bias about *having nightmares about the disease, not remembering the recommendations issued by the authorities, feeling worried about their general state of health and physical pain spots in the body, believing they were suffering from a severe physical disease (even though it had not been confirmed), and feeling like staying in bed, taking their temperature, taking their pulse, changing their diet, taking meds, etc. (even though they have not been prescribed by a physician).* According to COVID-19 status, such psychometric bias should be studied in future research despite considering high intrusion, avoidance, health anxiety, and somatization.

Our findings suggest that those seeking remote psychological care reported high levels of numbing and depression, which bias included *feeling distant from people with whom they had regularly interacted since the beginning of the pandemic, struggling to care about their loved ones*, and *wanting to hurt themselves.* The bias in such items should be addressed in the subsequent studies despite the accepted high levels of numbing and depression.

In keeping with Asmundson et al. (2000), Velasco et al. (2006), Goldberg et al. (2017), and Morales-Chainé et al. (2020, 2021a,2021b) in the present study, we screened mental health risks characterized by stress, depression, generalized anxiety, health anxiety, and somatization symptoms. Moreover, these symptoms were related to specific events such as losing loved ones, suspecting, or having COVID-19, and sociodemographic conditions such as sex. But the novel assumption was to show how those mental health symptoms were related to psychological care-seeking (Goldberg et al., 2017), through CFA analysis for measurement invariance.

Our findings indicate that it was possible to validate the factor structure of stress symptoms and their relationship with depression, anxiety, and somatization. We also described the specific bias as a function of sociodemographic conditions reported by participants during the COVID-19 pandemic. This identification was conducted in community and primary health care scenarios and with the CFA of the PCL, depression, generalized anxiety, health anxiety, and somatization scales, adapted for use during the COVID-19 pandemic (Morales-Chainé et al., 2020, 2021a,2021b).

Furthermore, in the future, researchers could monitor the process and the time elapsed between the occurrence of traumatic events and the development of PTSD, as well as other mental health risks, through measurement tools such as those used in this study. However, the present study suggested an association between these dimensions with depression, anxiety, and somatization. Already Elhai and Palmieri (2011) recommended studying such associations to better define the etiology and development of PTSD in the early stages.

The final model suggested that intrusion was predicted by avoidance symptoms. The health anxiety latent variable was predicted by intrusion stress symptoms (Morales-Chainé et al., 2021b). Moreover, generalized anxiety, the latent variable with higher levels in women, those suspected of having or being infected with COVID-19, and those who sought remote psychological care, were predicted by health anxiety and hyperactivation symptoms. The hyperactivation latent variable, as a stress symptom, was predicted by depression symptoms. According to the final model, depression and somatization were predicted by health anxiety. Finally, the numbing latent variable was predicted by depression and avoidance ones. As a result of the high prevalence of stress, anxiety, and depression in the global world related to the COVID-19 pandemic, it is essential to screen for these mental health conditions at the community and primary health care level (Necho et al., 2021).

This sequence of symptoms could help predict more severe disorders. Consequently, the programmed tools helped identify depression early, together with anxiety symptoms, as Goldberg et al. (2017) established. They proposed that depression was closely associated with anxiety symptoms. Authors noted that anxious depression could be the most common comorbidity, helping to predict more severe disorders at the community level. Goldberg et al. (2017) proposed that anxious depression (high scores on both scales) could be the most common risk in people seeking specialized or regular care. However, early identification of mental health symptoms could be considered in terms of the common variance between different emotional disorders to monitor continuity in the anxiety and depression case-no-case progression. Therefore, Goldberg et al. (2017) considered it essential to establish screening in each territorial entity, interpreting the results by each specific community context.

Moreover, in that sequence of symptoms, the SSCOs were strongly related to anxiety and depression, suggesting that SSCOs were successfully screened by sex, COVID-19 status, loss of loved ones, and seeking remote psychological care. According to Velasco et al. (2006), SSCO etiology and maintenance could be related to lifestyles, learning, beliefs, and antisocial behaviors that could be related to the COVID-19 pandemic. Further studies should address the specific processes explaining these conditions and relationships.

Nevertheless, in the current research, it was possible to develop a decision-making strategy due to the tool’s psychometric characteristics and the stress, depression, anxiety, and somatization latent variables, in Mexico. These conditions could vary by country, clinical scenario, and population characteristics (Goldberg et al., 2017). However, it is possible to detect a psychological disorder efficiently and early in the community and provide the necessary primary care by monitoring these symptoms (Moos, 1995).

Furthermore, the measurement invariance procedure, suggested by McDonald and Calhoun (2010) and Elhai and Palmieri (2011), ensured the detection of specific biases from symptoms and comparison groups (Millsap, 2011), which is essential to consider when specific populations and disturbing phenomena are analyzed. The identification of bias addresses decision-making because mental health symptoms vary depending on the context (Wilkins et al., 2011). CFA ensured evidence about the psychometric structure of the scales through the assessment of the measurement invariance between the comparison groups in this study. A significant contribution of this study was the measurement invariance examination. This specific contribution is a requirement for establishing a valid comparison between groups by latent variables rather than the differences in the psychometric structure of the scale items.

Moreover, future studies should describe posttraumatic stress diagnosis, assessing the cut-off points in the PCL’s intrusion, avoidance-numbing, and hyperactivation symptoms. It could discriminate between the presence and absence of stress levels, as Taylor et al. (1998) and McDonald and Calhoun (2010) recommended. Future studies could help distinguish between anxiety and somatization levels (Velasco et al., 2006; Goldberg et al., 2017; Morales-Chainé et al., 2020, 2021a,2021b) when experiencing events such as the COVID-19 pandemic.

The PCL, depression-generalized anxiety, and health anxiety-somatization scales are a realistic group of descriptions for early, parsimonious mental health symptom screening in community and primary health care services. Implementing effective evidence-based psychosocial interventions would be helpful to reduce the care gap and promote mental health (Li et al., 2020). Rather than a diagnostic strategy, the early screening of mental health symptoms (McDonald and Calhoun, 2010) is a tool for achieving efficient programming, resulting from a step-by-step, evidence-based intervention, given the lack of specialized professionals in Latin American countries.

## Limitations

Since the present study was not a diagnosis of mental health disorders, future studies should ensure their follow-up and assess consistency with these diagnoses and evaluate the effect of remote psychological help. Since this study is not longitudinal, in the future, researchers could monitor the process and the time that has elapsed between the occurrence of traumatic events and the development of a posttraumatic stress disorder, as well as other mental health risks, through measurement tools such as those used in this study.

One limitation referred to bias. We considered it necessary to study the sources of the bias from the items identified through the invariance measurement and unexplained variance from the SEM. Moreover, note that we did not assess the age as a confounder in the data analyses, and our sample was not homogeneous because the participation was voluntary. Thus, evaluating invariance due to age groups as a confounder would help identify other bias origins when such wide variations of participants’ age are like those considered here. Next, studies should consider age groups to assess invariance measurement. Dynamically, identifying the source of bias would make it possible to increase the accuracy of mental health symptom screening and halt the evolution of mental illness.

Another limitation refers to psychometrical considerations. CFA was a useful defining factor structure of the mental health tools. Invariance measurement helped analyze how individuals responded to items, and SEM helped identify the unexplained variances from latent variables. Even though, we should carefully consider the results from the use of the Cronbach Alpha analysis. We didn’t study the uncorrelated errors among items and the effect of violating this assumption on alpha (Green and Hershberger, 2000). Thus, such conditions produce an unprecise high estimate of reliability that must be considered in future studies.

Additionally, we must consider a strategy to increase the representativeness of our sample to analyze mental health symptoms. Because participants voluntarily chose to contribute, we could not achieve this condition. Finally, subsequent studies should consider social determinants during the COVID-19 pandemic, such as age, unemployment, intra-familial violence, and the use of drugs such as alcohol and tobacco, to understand how they contribute to the early emergence of mental health symptoms.

## Data Availability Statement

The original contributions presented in the study are included in the article/Supplementary Material, further inquiries can be directed to the corresponding author.

## Ethics Statement

The studies involving human participants were reviewed and approved by Ethics Committee of the Universidad Nacional Autónoma de México. The participants provided their written informed consent to participate in the study.

## Author Contributions

SM and MR contributed to the writing and initial data analysis. SM, RR, AL, ABM, ABA, CT, GP, IL, LB, and MR contributed to the data analysis review, discussion, and data interpretation. All authors contributed to the article and approved the submitted version.

## Conflict of Interest

The authors declare that the research was conducted in the absence of any commercial or financial relationships that could be construed as a potential conflict of interest.

## Publisher’s Note

All claims expressed in this article are solely those of the authors and do not necessarily represent those of their affiliated organizations, or those of the publisher, the editors and the reviewers. Any product that may be evaluated in this article, or claim that may be made by its manufacturer, is not guaranteed or endorsed by the publisher.

## References

[B1] American Psychiatric Association [APA] (2000). *Diagnostic and Statistical Manual of Mental Disorders*, 4th Edn. Washington, DC: Autor.

[B2] American Psychiatric Association [APA] (2013). *Diagnostic and Statistical Manual of Mental Disorders*, 5th Edn. Washington, DC: Autor.

[B3] Arrieta J. Aguerrebere M. Raviola G. Flores H. Elliott P. Espinosa A. (2017). Validity and utility of the patient health questionnaire (PHQ)-2 and PHQ-9 for screening and diagnosis of depression in Rural Chiapas, Mexico: a cross-sectional study. *J. Clin. Psychol.* 73 1076–1090. 10.1002/jclp.22390 28195649 PMC5573982

[B4] Asmundson G. J. G. Frombach I. McQuaid J. Pedrelli P. Lenox R. Stein M. B. (2000). Dimensionality of posttraumatic stress symptoms: a confirmatory factor analysis of DSM-IV symptom clusters and other symptom models. *Behav. Res. Ther.* 38 203–214. 10.1016/s0005-7967(99)00061-3 10661004

[B5] Browne M. W. Cudeck R. (1993). “Alternative ways of assessing model fit,” in *Testing Structural Equation Models*, eds Bollen K. A. Long J. S. (Newbury Park, CA: Sage Publiations), 136–162.

[B6] Cheug G. W. Rensvold R. B. (2002). Evaluating goodness-of-fit indexes for testing measurement invariance. *Struct. Equ. Model.* 9 233–255. 10.1207/s15328007sem0902_5 33486653

[B7] Elhai J. D. Palmieri P. A. (2011). The factor structure of posttraumatic stress disorder: a literature update, critique of methodology, and agenda for future research. *J. Anxiety Disord.* 25 849–854. 10.1016/j.janxdis.2011.04 21793239

[B8] Goldberg D. P. Reed G. M. Robles R. Minhas F. Razzaque B. Fortés S. (2017). Screening for anxiety, depression, and anxious depression in primary care: A field study for ICD-11 PHC. *J. Affect. Disord.* 213 199–206. 10.1016/j.jad.2017.02.025 28278448

[B9] Green S. B. Hershberger S. L. (2000). *Structural Equation Modeling*, Vol. 7. Mahwah, NJ: Lawrence Erlbaum Associates, 251–270.

[B10] Jöreskog K. G. (1971). Statistical analysis of sets of congeneric tests. *Psycometrika* 36 109–133. 10.1007/BF02291393

[B12] Li S. Wang Y. Xue J. Zhao N. Zhu T. (2020). The impact of COVID-19 epidemic declaration on psychological consequences: a study on active Weibo users. *Int. J. Environ. Res. Public Health* 17:2032. 10.3390/ijerph17062032 32204411 PMC7143846

[B13] McDonald S. D. Calhoun P. S. (2010). The diagnostic accuracy of the PTSD Checklist: a critical review. *Clin. Psychol. Rev.* 30 976–987. 10.1016/j.cpr.2010.06.012 20705376

[B14] Millsap R. E. (2011). *Statistical Approaches to Measurement Invariance.* New York, NY: Routledge, 10.4324/9780203821961

[B15] Moos R. H. (1995). Development and applications of new measures of life stressors, social resources and coping responses. *Eur. J. Psychol. Assess.* 11 1–13. 10.1027/1015-5759.11.1.1

[B16] Morales-Chainé S. López M. A. Bosch M. A. Beristain A. A. Robles G. R. López R. F. (2020). Condiciones de salud mental durante la pandemia por COVID-19. *Rev. Int. Investig. Adicciones* 6 11–24. 10.28931/riiad.2020.2.03

[B17] Morales-Chainé S. López M. A. Bosch M. A. Beristain A. A. Robles G. R. Garibay R. C. R. (2021a). Mental health symptoms, binge drinking, and the experience of abuse during the COVID-19 Lockdown in Mexico. *Fronti. Public Health* 9:656036. 10.3389/fpubh.2021.656036 34368044 PMC8342039

[B18] Morales-Chainé S. López M. A. Bosch M. A. Beristain A. A. Escobar G. G. Robles G. R. (2021b). Condiciones socioeconomicas y de salud mental durante la pandemia por COVID-19. *Acta Investig. Psicol.* 11 5–23. 10.22201/fpsi.20074719e.2021.2.379

[B19] Necho M. Tsehay M. Birkie M. Biset G. Tadesse E. (2021). Prevalence of anxiety, depression, and psychological distress among the general population during the COVID-19 pandemic: a systematic review and meta-analysis. *Int. J. Soc. Psychiatry* 67 892–906. 10.1177/00207640211003121 33794717

[B20] Panamerican Health Organization (2022). *PAHO COVID-19 Response.* Available online at: arcgis.com (accessed March 29, 2022).

[B21] Rogers J. P. Chesney E. Oliver D. Pollak T. A. McGuire P. Fusar-Poli P. (2020). Psychiatric and neuropsychiatric presentations associated with severe coronavirus infections: a systematic review and meta-analysis with comparison to the COVID-19 pandemic. *Lancet Psychiatry* 7 611–627. 10.1016/52215-0366(20)30203-0 32437679 PMC7234781

[B22] Secretaría de Salud en México (2020). *Information General Sobre Coronavirus (COVID-19).* Available online at: https://www.gob.mx/salud (accessed April 9, 2020).

[B23] Sörbom D. (1974). A general method for studying differences in factor means and factor structures between groups. *Br. J. Math. Stat. Psychol.* 27 229–239. 10.1111/j.2044-8317.1974.tb00543.x

[B24] Spanish Acronym LGPDPPSO (2017). *Ley General de Protección de Datos Personales en Posesión de Sujetos Obligados.* Available online at: http://www.diputados.gob.mx/LeyesBiblio/pdf/LGPDPPSO.pdf (accessed July 10, 2020).

[B25] Taylor S. Kuch K. Koch W. J. Crockett D. J. Passey G. (1998). The structure of posttraumatic stress symptoms. *J. Abnorm. Psychol.* 107 154–160. 9505048 10.1037//0021-843x.107.1.154

[B26] Vanderberg R. J. Lance C. E. (2000). A review and synthesis of the measurement invariance literature: suggestions, practices, and recommendations for organizational research. *Organ. Res. Method* 3 4–70. 10.1177/109442810031002

[B27] Velasco S. Ruíz M. T. Álvarez-Dardet C. (2006). Modelos de atención a los síntomas somáticos sin causa orgánica. De los trastornos fisiopatológicos al malestar de las mujeres. *Rev. Esp. Salud Pública* 80 317–333. 10.1590/s1135-57272006000400003 16913608

[B28] Weathers F. W. Litz B. T. Huska J. Keane T. (1994). *PTSD CheckList (PCL-C).* Boston, MA: National Center for PTSD-Behavioral Science Division.

[B29] West S. G. Taylor A. B. Wu W. (2012). “Model fit and model selection in structural equation modeling,” in *Handbook of Structural Equation Modeling*, ed. Hoyle R. H. (New York, NY: Guilford Press), 380–392.

[B30] Wilkins K. C. Lang A. J. Norman S. B. (2011). Synthesis of the psychometric properties of the PTSD checklist (PCL) military, civilian, and specific versions. *Depress. Anxiety* 28 596–606. 10.1002/da.20837 21681864 PMC3128669

